# 388. Evaluating Rates of Co- and Secondary Infections in Pediatric Patients with COVID-19 Infections in Relation to Antibiotic Prescribing: An Observational Study

**DOI:** 10.1093/ofid/ofad500.458

**Published:** 2023-11-27

**Authors:** Brian S Allen, Sarah E Firmani, Bonnie C Prokesch

**Affiliations:** University of Missouri, Columbia, Missouri; Children's Health, Watauga, Texas; Parkland Health, Dallas, Texas

## Abstract

**Background:**

All ages have been greatly impacted by the Coronavirus disease 2019 (COVID-19) pandemic. While many patients with COVID-19 are prescribed antibiotics, there is often little microbiologic evidence for concurrent or secondary bacterial infection. Such prescribing practices may lead to increased antimicrobial resistance. The purpose of this study is to evaluate the number of microbiologically confirmed bacterial co- and secondary infections in pediatric patients with COVID-19 and characterize associated antimicrobial use at a large academic medical center.

**Methods:**

An observational, retrospective review of pediatric patients ≤ 18 years admitted to Children’s Medical Center Dallas campus with COVID-19 infections from 5/01/2020 to 9/30/2022 was performed. Basic demographic, epidemiologic, laboratory, and microbiologic data was obtained upon admission and each new antibiotic course. Cultures and microbiologic data were reviewed for relevance. Antibiotic use was characterized based on drug selection, indication, and spectrum.

**Results:**

Using the International Classification of diseases, tenth revision (ICD-10) code for COVID-19 pneumonia (U07.1) identified 229 patients, of which 164 met inclusion criteria. Within the included patient population, a total of 22 microbiologically-proven infections were found. Both co- and secondary infections were rare (8/164 (5%) and 8/164 (5%), respectively) and consisted of various organisms from differing sites (9 pulmonary, 9 bloodstream, 4 urinary tract, 1 *Clostridiodes difficile* infection). The most frequently identified organism was *Staphylococcus aureus*, followed by *Escherichia coli* and *Pseudomonas aeruginosa*. 102/164 (63%) children received antibiotics ≤ 48 hours of admission. There were 43 additional instances where antibiotics were started > 48 hours after admission.

**Conclusion:**

Both co- and secondary bacterial infections are rare in pediatric patients with COVID-19 pneumonia, but many hospitalized patients are given antibiotics. Antibiotics were continued in the absence of microbiologic data potentially increasing antimicrobial resistance. There is clearly a role for antimicrobial stewardship in pediatric patients with COVID-19 pneumonia.

**Disclosures:**

**All Authors**: No reported disclosures

